# Long Non-Coding RNAs in Vascular Inflammation

**DOI:** 10.3389/fcvm.2018.00022

**Published:** 2018-03-14

**Authors:** Stefan Haemmig, Viorel Simion, Mark W. Feinberg

**Affiliations:** ^1^Cardiovascular Division, Department of Medicine, Brigham and Women’s Hospital, Harvard Medical School, Boston, MA, United States

**Keywords:** lncRNAs, vascular inflammation, cardiovascular disease, acute inflammation, chronic inflammation

## Abstract

Less than 2% of the genome encodes for proteins. Accumulating studies have revealed a diverse set of RNAs derived from the non-coding genome. Among them, long non-coding RNAs (lncRNAs) have garnered widespread attention over recent years as emerging regulators of diverse biological processes including in cardiovascular disease (CVD). However, our knowledge of their mechanisms by which they control CVD-related gene expression and cell signaling pathways is still limited. Furthermore, only a handful of lncRNAs has been functionally evaluated in the context of vascular inflammation, an important process that underlies both acute and chronic disease states. Because some lncRNAs may be expressed in cell- and tissue-specific expression patterns, these non-coding RNAs hold great promise as novel biomarkers and as therapeutic targets in health and disease. Herein, we review those lncRNAs implicated in pro- and anti-inflammatory processes of acute and chronic vascular inflammation. An improved understanding of lncRNAs in vascular inflammation may provide new pathophysiological insights in CVD and opportunities for the generation of a new class of RNA-based biomarkers and therapeutic targets.

## Introduction

Accumulating studies highlight that inflammatory processes and traditional cardiac risk factors may cooperatively contribute to vascular disease leading to the development of cardiovascular events (1). A variety of systemic inflammatory diseases such as rheumatoid arthritis (RA), systemic lupus erythematosus, psoriatic arthritis, and medium to large vessel vasculitis are associated with an increased risk of atherosclerotic events and premature cardiovascular disease (CVD) (2). Interestingly, acute inflammation (e.g., sepsis) also significantly increases the risk of future cardiovascular events (3). Although these diseases differ in their autoimmune and/or inflammatory nature, atherosclerosis may represent a common response with local vascular inflammation in subintimal and perivascular layers. Over decades, a progressive inflammatory multistep process in lesion-prone regions of the arterial vasculature develops by different disease-specific upstream insults (1, 4). However, our understanding of the pathophysiological links between systemic inflammatory diseases to vascular inflammation remains poorly understood. The recent recognition that as much as 70–90% of the genome is pervasively transcribed at some point during development has opened new opportunities to address these questions (5–7). Most of those transcripts are non-coding measuring greater than 200 nucleotides in length and display mRNA-like processing properties. This class of non-coding RNA (ncRNA) is known as long ncRNAs (lncRNAs). Whereas the number of ~19,000 human protein-coding genes has plateaued, the number of lncRNAs keeps increasing annually. However, it should be pointed out that lncRNAs have a low cross-species conservation rate and many lncRNAs have extremely low expression levels per cell (8–10). Nevertheless, lncRNAs have emerged as powerful regulators of nearly all biological processes by mediating epigenetic, transcriptional, or translational control of target genes due to their polyvalent binding properties to RNA, DNA, and protein as well as acting as molecular sponges for other transcripts and miRNAs (11). The subcellular localization pattern can provide additional insights into the mechanistic role for lncRNAs. Other considerations include whether the lncRNA acts in *cis* or *trans* and whether the RNA product itself is essential for fulfilling its function or if its transcription *per se* that underlies its function (12). However, the role of lncRNAs in vascular inflammation and CVD is just emerging (13). This review will summarize recent findings that provide mechanistic and translational insights of lncRNAs in acute and chronic vascular inflammation in the context of CVD.

## Acute Inflammation

The acute inflammatory response is induced as a first line of defense against microbial infection and other “non-self” stimuli. Antigen-presenting cells express different receptors, of which the Toll-like receptor (TLR) family is best characterized. TLRs are especially sensitive to microbes products such as LPS, lipoproteins, and nucleic acids (14). Once activated, these receptors trigger complex signaling cascades resulting in changes in expression of hundreds of genes involved in immunity and inflammation. TLRs have been implicated in destabilizing plaques leading to atherothrombosis in the vessel wall. For example, TLR4 enhances macrophage responses to lipids and inflammation, whereas TLR2 potentiates inflammation more broadly in the vessel wall in both macrophages and vascular cells, an effect that may lead to superficial erosion of atherosclerotic lesions (15, 16). Recent studies have connected a range of acute inflammatory processes to lncRNA expression and found that lncRNAs can regulate the acute inflammatory response, opening new avenues for exploring pathophysiological insights that may lead to improved disease stage-specific diagnostics and therapeutic interventions.

Metastasis-associated lung adenocarcinoma transcript 1 (*MALAT1*) is a conserved lncRNA whose expression correlates with many human cancers. Recent data also indicate its significance in immunity, and specifically in acute inflammation (17). *MALAT1* expression is increased in LPS-activated macrophages (17), cardiac microvascular endothelial cells (CMVEC), and in the hearts of rats with sepsis (18). Knockdown of *MALAT1* increases the LPS-induced expression of TNFα and IL-6 in macrophages. Mechanistically, *MALAT1* interacts with NF-κB in the nucleus to inhibit its DNA binding activity, and consequently decreases the production of inflammatory cytokines (17). *MALAT1* was also found to interact with the polycomb protein EZH2 in CMVECs in response to LPS activation (18). In a recent study, *MALAT1* expression was increased by LPS stimulation in murine cardiomyocytes and in cardiac tissue of a mouse sepsis model (19). *MALAT1* overexpression enhanced TNFα production on LPS-stimulated cardiomyocytes, while *MALAT1* siRNA had an inhibitory effect, *via* serum amyloid antigen 3 (SAA3), an inflammatory ligand that can stimulate IL-6 and TNFα production, as observed in other cells such as endothelial (20) and mouse liver cells (21). Cardiomyocytes transfected with *MALAT1* siRNA were less susceptible to LPS-induced cell apoptosis, suggesting that *MALAT1* induction is a mechanism of cardiomyocyte apoptosis or injury in response to sepsis (19). Collectively, *MALAT1* differentially regulates inflammatory responses in a cell-specific manner.

*lincRNA-Cox2*, is a lncRNA neighboring the Ptgs2 (Cox2) gene, recently discovered as a key regulator of inflammatory responses mediating both the activation and repression of distinct classes of immune genes (22). LPS stimulation induced *lincRNA-Cox2* expression in both dendritic cells and bone-marrow derived macrophages (BMDM) in a similar pattern as Ptgs2 (22, 23). *lincRNA-Cox2* expression is induced by LPS in a MyD88- and NF-κB–dependent manner and *lincRNA-Cox2* silencing/overexpression in BMDM regulates important immune genes such as TNFα, IL-6, CCL5, SOCS3, and STAT3 (22). Several mechanisms have been described for *lincRNA-Cox2*, including interaction with heterogeneous nuclear ribonucleoprotein (hnRNP) A/B and A2/B1 (22), degradation of IKB-α in the cytosol, and assembly into the SWItch/Sucrose NonFermentable (SWI/SNF) complex, thereby acting as a co-activator of NF-κB or inducing SWI/SNF-associated chromatin remodelling (24, 25). In a recent study, *lincRNA-Cox2* modulated TNFα–induced transcription of the IL-12b gene by promoting the recruitment of Mi-2/nucleosome remodeling and deacetylase (Mi-2/NuRD) repressor complex to the IL-12b promoter region (26). Taken together, rapid activation of *lincRNA-Cox2* may regulate a range of acute inflammatory signaling pathways.

*THRIL* (TNFα and hnRNPL related immune-regulatory LincRNA) or *linc1992,* is a lncRNA that regulates TNFα expression through a negative feedback mechanism. *THRIL* binds specifically to heterogenous nuclear ribonucleoprotein L (hnRNPL) and forms a functional *THRIL*–hnRNPL complex that regulates transcription of the TNFα gene by binding to its promoter. *THRIL* is also required for expression of many immune-response genes and regulators of TNFα expression. Clinically, *THRIL* expression correlated with the severity of symptoms in patients with Kawasaki disease, an acute inflammatory disease of childhood (27). This study provides strong evidence that *THRIL* is required for induction of TNFα expression and plays an important role in acute inflammation and innate immunity. Further studies will be of interest to verify these findings in other inflammatory contexts, including chronic inflammation such as atherosclerosis or diabetes.

## Chronic Inflammation

Chronic inflammation is a major contributing factor to vascular events, including atherosclerotic plaque development, plaque erosion, aortic aneurysm, and ischemic myocardial damage. Inflammation disturbs the homeostasis of the endothelium, leading to endothelial dysfunction, which is amongst the earliest processes involved in atherosclerotic initiation (28). The early response is characterised by activation of endothelial cells (ECs), triggered by biochemical (e.g., IL-1β, TNFα, oxLDL, etc.) and biomechanical stimulation in the form of disturbed blood flow [Rev by (29)]. Consequently, expression of adhesion molecules (e.g., VCAM-1, ICAM-1, E-Selectin) and secretion of membrane-associated chemokines (e.g., MCP-1, fractalkine) fosters the recruitment of monocytes and different types of T cells to the vessel wall (14–17). Chronic endothelial activation leading to the loss of endothelial integrity increases the risk for atherosclerosis. This is often observed in patients with RA, an autoimmune disease that causes chronic inflammation of the joints and systemically in the vasculature (30). A strong relationship exists between RA and atherosclerosis, but causality remains unclear.

Spurlock et al. identified that the expression of the lncRNA *lincRNA-p21* is significantly lower specifically in patients with RA compared to healthy subjects. No dysregulation of *lincRNA-p21* could be observed in systemic lupus erythematosus and Sjörgen’s syndrome (31). Interestingly, *lincRNA-p21* could be restored to normal levels in RA patients treated with methotrexate (MTX), which is the most commonly used anti-rheumatic drug with anti-inflammatory properties (32). *In vitro* analysis of Jurkat T cells confirmed the induction of *lincRNA-p21* by MTX (31). Initial work discovered *lincRNA-p21* as a repressor of p53-dependent transcriptional responses. Silencing of *lincRNA-p21* affected the expression of hundreds of genes known to be repressed by p53, which could be rescued by inhibiting p53, suggesting that *lincRNA-p21* functions as a downstream repressor for p53. This transcriptional repression by *lincRNA-p21* is mediated through an interaction with hnRNP-K (33). Because p53 expression levels positively correlated with *lincRNA-p21* expression in RA patients, basal *lincRNA-p21* expression may be p53-independent in PBMCs. In addition, *lincRNA-p21* was initially described to be regulated by p53 in the context of DNA damage response (33). To investigate whether specific inhibition of either ATM or DNA-PKcs, two key upstream regulators of the DNA damage response (34, 35), could restore *lincRNA-p21* or p53 expression, inhibition studies were performed. Indeed, MTX-mediated induction of p53 *and lincRNA-p21* was blocked in Jurkat T cells treated with NU-7441 (i.e., inhibitor for DNA-PKcs), whereas there was no effect using low concentration of KU-55933 (inhibitor for ATM). Furthermore, using *in vitro* NF-κB luciferase reporter assays, silencing of *lincRNA-p21* abrogated MTX-mediated inhibition of NF-κB activity. This effect could be simulated by using the NU-7441 inhibitor, demonstrating a link between *lincRNA-p21* and DNA-PKcs-mediated regulation of NF-κB pathway (31). This finding is consistent with previous reports, demonstrating that DNA-PKcs is a regulator of inflammation by phosphorylating p50, a member of the NF-κB pathway (36). Collectively, these findings suggest that MTX decreases the NF-κB pathway by increasing *lincRNA-p21* levels through a DNA-PKcs-dependent mechanism (31).

Stuhlmüller et al. described high expression levels of the lncRNA *H19* in synovial tissues and isolated synovial macrophages or synovial fibroblasts (SFB) from donor samples of RA patients compared to control subjects. *H19* was also induced in SFB from RA *ex vivo* using multiple pro-inflammatory stimuli such as TNFα, IL-1β, or PDGF-BB. Whether elevated levels of *H19* in RA reflects its role as a biomarker of inflammatory stimuli or as a pathogenic mediator remains unknown (37, 38). Future studies will be required to further define the functional role of lncRNA *H19* in RA pathogenesis and CVD.

Genome wide associated studies (GWAS) have identified the *INK4b-ARF-INK4a* locus located on chromosome 9p21 with multiple single nucleotide polymorphisms (SNPs) linked to coronary artery disease (CAD) (39–41), atherosclerosis (42), aortic aneurysm (43), ischemic stroke (41), type II diabetes (44) as well as specific cancer subtypes (45, 46). The lncRNA *ANRIL* (Antisense Non-coding RNA in the INK4 Locus) lies in opposite direction to the *INK4b-ARF-INK4b* locus, which contains the critical tumour suppressor genes p14^ARF^, p15^INK4b^ and p16^INK4a ^(47). The SNPs associated with CAD do not correlate with well-established CAD risk factors, suggesting that this lncRNA is a novel independent driver for vascular inflammation. Specifically two *ANRIL* transcripts (EU741058 and NR_003529) are significantly increased from patients with CAD in human atherosclerotic plaque tissue as well as peripheral blood mononuclear cells, whereas the most abundant isoform DQ485454 is not differentially expressed (48). Loss-of-function studies reduced cell viability of SMCs for siRNAs targeting exclusively NR_003529 or both NR_003529 and DQ485454 isoforms (49). Moreover, *ANRIL* silencing increased the expression level of the antisense transcripts p15^ARF^ and p16INK4b, both key regulators for senescence, apoptosis, and stem cell self-renewal by the retinoblastoma-p53 pathway abrogating PRC-1/2 binding to their loci (50). Additionally, *ANRIL* binds directly to PRC-1/2 components (i.e., CBX7 and/or SUZ12) supporting its role in regulating epigenetics (51). The multiple splice sites of *ANRIL* may result in isoform-specific effects, thus explaining some paradoxical findings. For example, an interesting isoform is circular *ANRIL* (*cANRIL*), which results from exon skipping events during RNA splicing (52), adding another layer of complexity for the biological understanding of the *ANRIL* locus. *cANRIL* binds to PES1, an essential 60S-preribosomal assembly factor, impairing pre-rRNA processing and ribosome biogenesis in SMCs and macrophages. As a consequence, *cANRIL* induces nucleolar stress and p53 activation, resulting in the inhibition of proliferation and induction of apoptosis, as observed for the linear *ANRIL* (53). Although *ANRIL* is an independent risk factor for CAD, its functional role in vascular inflammation in CVD still requires clarity based upon transcript specificity.

Hu et al. (54) identified increased expression of the lncRNA *RP5-833A20.1* in human foam cells. Gain-of-function studies demonstrated that *RP5-833A20.1* reduced cholesterol efflux and increased inflammatory cytokines, including IL-1β, IL-6, and TNFα in THP-1 macrophages. Mechanistically, *RP5-833A20.1* decreased the expression of NFIA by inducing miR-382–5 p expression. However, the specific mechanism of how *RP5-833A20.1* regulates miR-382–5 p expression for macrophage foam formation and verification of these findings *in vivo* will require further investigation (54).

Recently, Tontonoz et. al. demonstrated that *in vivo* delivery of the liver-expressed liver X receptor-induced lncRNA (*LeXis*) reduced aortic lesion size by Oil-red O staining (55). Since *LeXis* has been previously described to maintain hepatic sterol content and levels of serum cholesterol (56), the adenovirus-mediated *LeXis* overexpression in the liver was specifically designed using a thyroxine-binding globulin promoter. In line with their previous findings, *LeXis* overexpression reduced total serum cholesterol levels (55). This study raises the possibility for long-term lncRNA therapy in mice. Future studies that can overexpress lncRNAs in the liver or vessel wall may provide a novel therapeutic approach for regulating vascular inflammation in CVD.

Recent studies have illustrated increased *HOTAIR* expression in PBMCs and serum exosomes of RA patients, while lower expression of *HOTAIR* was detected in differentiated osteoclasts and rheumatoid synoviocytes. Overexpression of *HOTAIR* using lentivirus decreased the expression of IL-17, IL-23, IL-1β, and TNFα, and inhibited the activation of NF-κB in LPS-treated chondrocytes in a miR-138 regulated manner (57). These findings are in line with a previous exploratory study where *HOTAIR* expression was significantly reduced in LPS-treated chondrocytes and a RA mouse model (58). However, findings from acute inflammatory states such as sepsis are opposite. Using a mouse model of sepsis, *HOTAIR* expression was significantly increased in cardiomyocytes. *HOTAIR* silencing improved the cardiac function of septic mice, and markedly decreased TNFα production in the circulation and p65 phosphorylation in cardiomyocytes (59). These studies highlight a cell-type dependent role for *HOTAIR* in acute inflammation. Future studies will be required to identify potential compensatory mechanisms that may be activated in cardiovascular cell types versus chondrocytes after LPS activation.

## Conclusions and Future Directions

Despite current prevention interventions and guideline-based therapeutics, recurrent cardiovascular events after acute coronary syndromes remain elevated at ~10% of patients within one year and ~20% of patients within 36 months of initial presentation (60, 61). Biomarkers for systemic inflammation such as high sensitivity C-reactive protein (hsCRP) has been associated with increased risk for cardiovascular events (62). Although the role of inflammation in atherosclerosis has been identified over 150 years ago by Virchow (63), only recently has the “inflammation hypothesis” in atherosclerosis been specifically tested with an anti-inflammatory drug targeting IL-1β. In the canakinumab anti-inflammatory thrombosis outcomes study (CANTOS), over 10,000 patients with elevated hsCRP at least 1 month post-myocardial infarction were randomized to receive the humanized monoclonal antibody canakinumab to neutralize IL-1β or placebo on top of usual therapy including statins. Impressively, recurrent cardiovascular events were reduced in the canakinumab treatment group independent of changes in lipid levels (64). As statin therapy lowers both LDL-C and inflammation (measured by hsCRP), CANTOS is the first clinical trial showing that lowering inflammation alone, without lowering LDL-C, significantly reduces cardiovascular events. Additional ongoing clinical trials using other anti-inflammatory drugs will likely provide further insights and impact clinical decision-making. For example, MTX is a widely used anti-inflammatory to treat RA patients (32). The cardiovascular inflammation reduction trial (CIRT) trial is currently investigating whether low dose of MTX administration will reduce the risk of cardiovascular events in patients with prior myocardial infraction and either type 2 diabetes or metabolic syndrome, all associated with chronic inflammation (ClinicalTrials.gov Identifier: NCT01594333) (65). Finally, the colchicine cardiovascular outcomes trial (COLCOT) will evaluate the long-term treatment of whether colchicine reduces rates of cardiovascular events in patients after myocardial infarction (ClinicalTrials.gov Identifier: NCT02551094).

**Figure 1:**
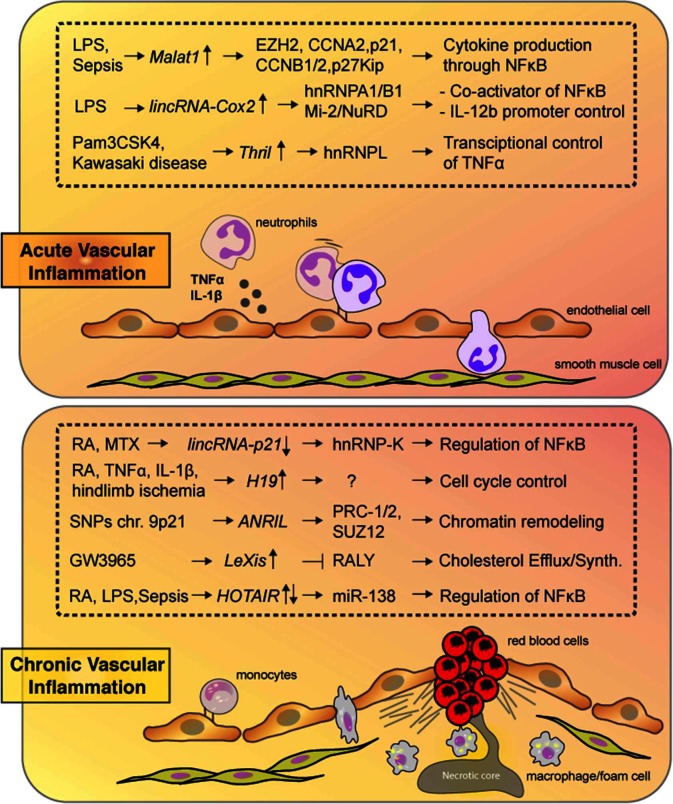
Highlighted lncRNAs from the text are shown along with their targets and biological consequences in response to acute (top) or chronic (bottom) vascular inflammation.

Our understanding of the estimated 50,000 human lncRNAs in regulating acute and chronic inflammatory processes in the vasculature remains nascent, although accumulating studies demonstrate that lncRNAs hold great promise as important regulators of vascular inflammation. Apart from their expression profile, functional *in vivo* findings are key to understand their true translational value in acute and chronic inflammation of the vasculature and links with cardiovascular disease states (Figure 1). Furthermore, emerging technical advances provide the ability to uncover novel lncRNA interactors (12). *ANRIL* represents a SNP-associated loci, which may bear relevance for CVD and other diseases. (39, 66). Sensitive biomarkers have emerged for chronic inflammation burden such as CRP, SAA, and IL-6 or vascular injury such as sICAM-1, sVCAM-1 and PTX3 (67). However, these two types of biomarkers tend to correlate weakly with each other. Because the expression of some lncRNAs track with stage-specific pathophysiological processes and they can be measured in serum (68), lncRNAs provide new avenues for diagnostics. For example, distinct lncRNAs may shed light on inflammatory subsets associated with systemic autoimmune diseases (e.g., RA, SLE) versus inflammation localized to the vessel wall (e.g., coronary or peripheral artery disease). Recent screening efforts of plasma from patients with CAD revealed a lncRNA named CoroMarker (AC100865.1) that was significantly increased in CAD patients compared to controls (69, 70). It will be of interest to examine whether this lncRNA is specific to CAD or increased in other chronic inflammatory diseases. While their translational value remains to be elucidated, ncRNA-based targeting strategies such as using antisense oligonucleotides (ASO) have already been approved for food and drug administration-approved drugs. Similar to miRNAs, lncRNAs are often differentially regulated in a cell-specific manner or in response to specific pathophysiological stimuli providing unique properties for therapeutic intervention. Challenges remain with the efficiency and specificity of delivery, which may be overcome by chemical modifications and/or nanoparticle carriers (71, 72). For example, ASOs that target liver-specific ligands [e.g., the liver-specific asialoglycoprotein receptor (ASGPR)] appear to confer strong efficacy and reasonable safety (73). Analogous paradigms for ASOs targeting vascular-specific ligands could provide novel therapeutics for vascular inflammation.

Collectively, because lncRNAs provide a new layer of control of protein-coding genes, lncRNAs may hold promise for uncovering novel pathophysiological insights, stage-specific biomarkers, and new targets for vascular inflammation in acute and chronic disease states.

## Author Contributions

SH and VS researched the data for the article, and significantly contributed to content and writing of the article. MF contributed to the conception of the article, and the writing, reviewing and editing of the draft manuscript.

## Conflict of Interest Statement

The authors declare that the research was conducted in the absence of any commercial or financial relationships that could be construed as a potential conflict of interest.
